# Supplementary figures and images for: Single cell resolution of SARS-CoV-2 tropism, antiviral responses, and susceptibility to therapies in primary human airway epithelium
Source: PLoS Pathog. 2021 Jan 28;17(1):e1009292. doi: 10.1371/journal.ppat.1009292 (PMC7872261; doi:10.1371/journal.ppat.1009292)

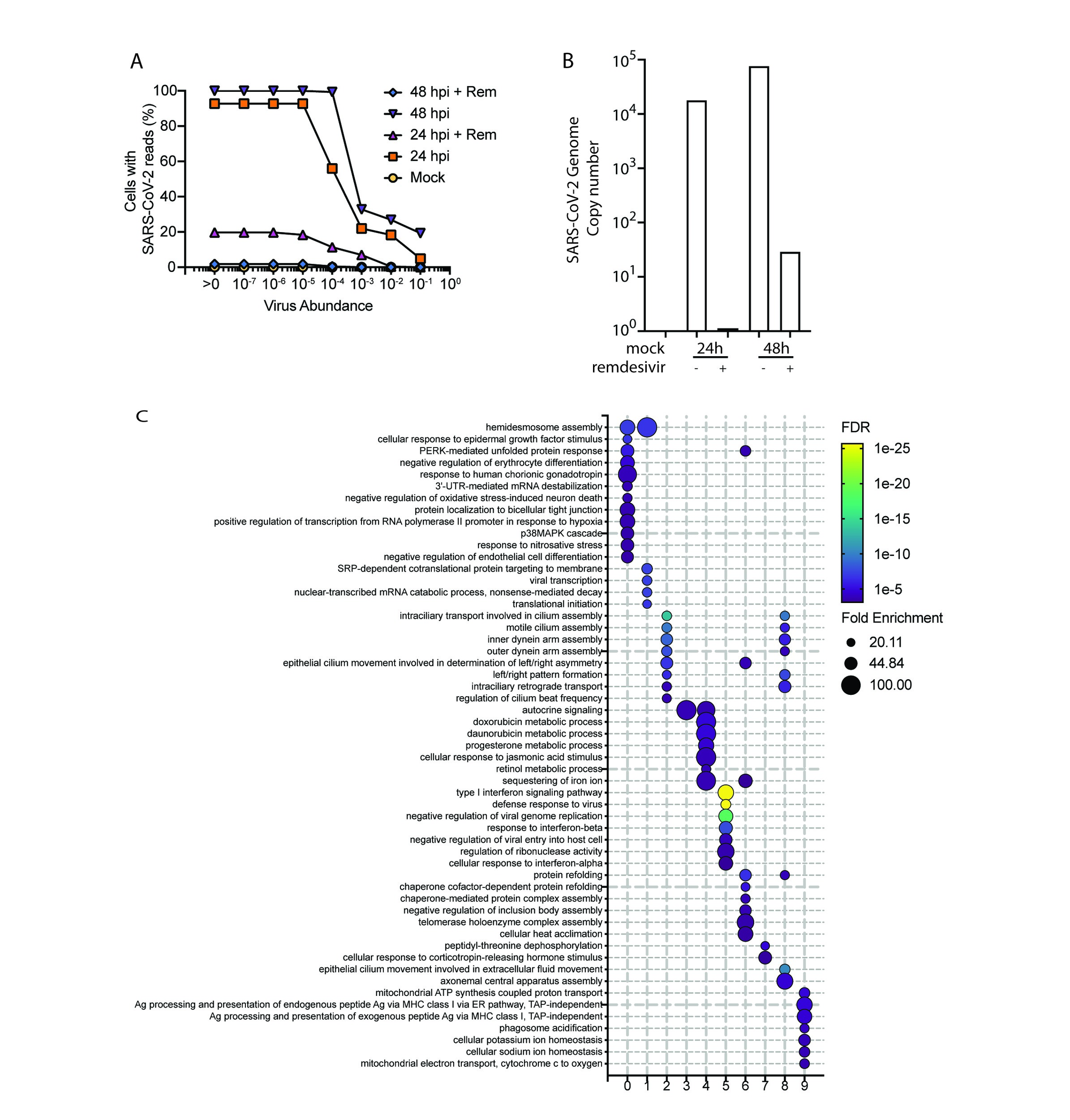

Supplement: S1 Fig — (A) Frequency of cells with the indicated percent of total reads mapping to SARS-CoV-2. (B) Total RNA from cells used in Fig 2 analyzed for SARS-CoV-2 (log10(RNA copies per sample)) detected in cells samples collected at 24 or 48 hours after infection with SARS-CoV-2 in the presence or absence of remdesivir calculated by qRT-PCR. (C) Gene ontology analysis of genes in each cluster with an adjusted p value <0.01 and LogFC >0.5 using Panther. Plotted GO terms had FDR <0.01 and FE >20. (TIF) [file ppat.1009292.s001.tif]

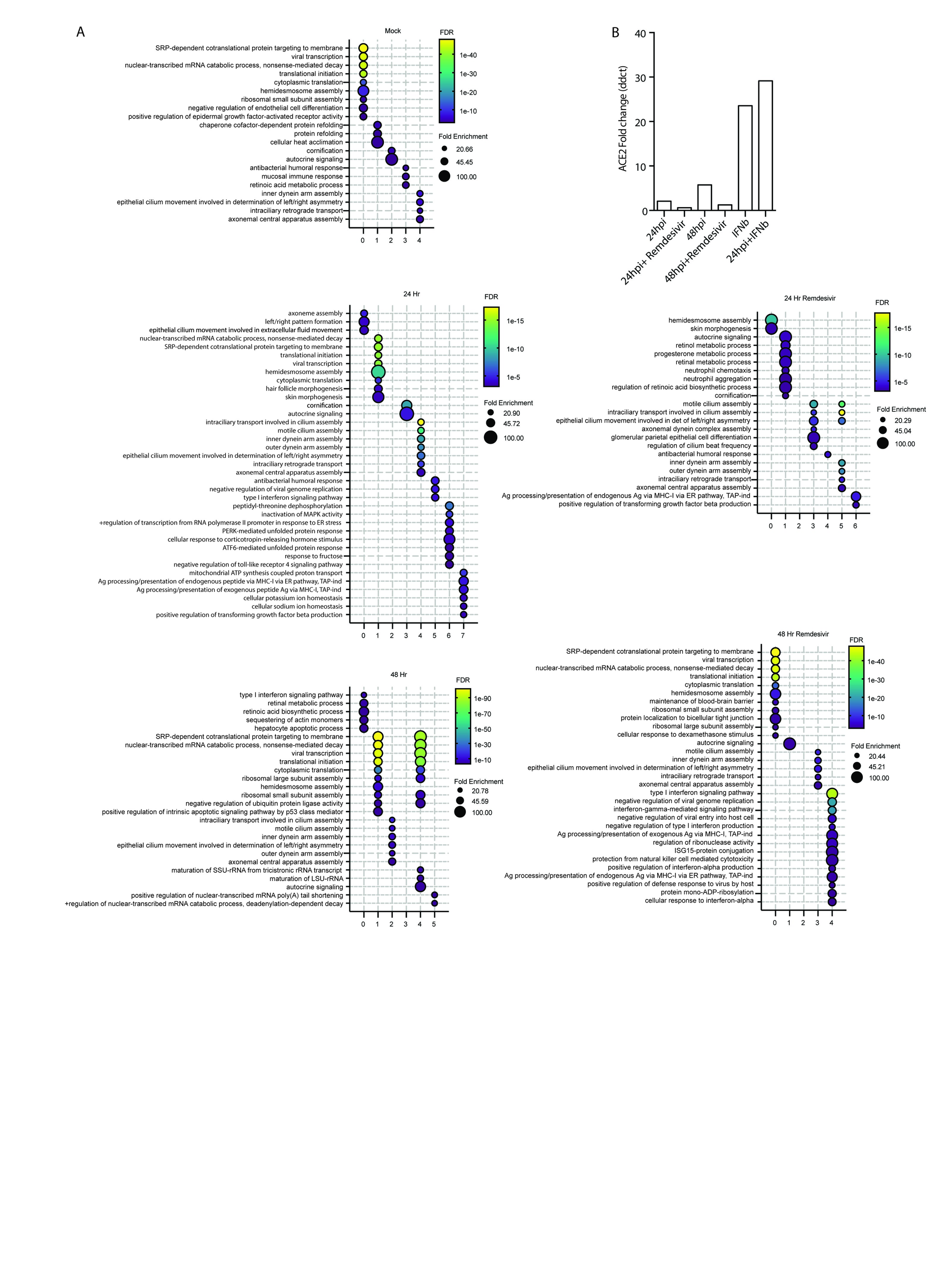

Supplement: S2 Fig — (A) Gene ontology analysis of genes in each cluster with an adjusted p value <0.01 and LogFC >0.5 using Panther. Plotted GO terms had FDR <0.01 and FE >20. (B) Total RNA from cells in Fig 2 and cells treated with or without IFNβ analyzed by qRT-PCR for ACE2. (TIF) [file ppat.1009292.s002.tif]
